# Inferring effective computational connectivity using incrementally conditioned multivariate transfer entropy

**DOI:** 10.1186/1471-2202-14-S1-P337

**Published:** 2013-07-08

**Authors:** Joseph T Lizier, Mikail Rubinov

**Affiliations:** 1 CSIRO Information and Communications Technology Centre, Marsfield, NSW 2122, Australia; 2 Max Planck Institute for Mathematics in the Sciences, 04103 Leipzig, Germany; 3 Department of Psychiatry, University of Cambridge, Cambridge, UK; 4 Churchill College, University of Cambridge, Cambridge, UK

## 

*Effective connectivity analysis *is a popular approach in computational neuroscience, whereby one seeks to infer a network of directed edges between neural variables (e.g. voxels or regions in fMRI, or reconstructed source time series in MEG data) which can explain their observed time-series dynamics. This is an important approach in understanding brain function, contrasting with functional connectivity analysis in being directed and dynamic, and with structural connectivity analysis in not requiring interventions and in being task-modulated. In particular, effective connectivity analysis seeks to find a *minimal *circuit model that can reconstruct the activity patterns contained in the given data. Ideally, such inference would be: made using model-free techniques; capture non-linear, multivariate, directional relationships; handle small amounts of data, and be statistically robust.

The information-theoretic measure *transfer entropy *(TE) [1] (a non-linear Granger causality) is becoming widely used for this purpose [2]. However its use is generally focussed only on interactions between a *single *source and destination (inferring an edge where TE is statistically significant), or else attempting multivariate considerations by *conditioning on all other *variables in the system (only practically possible with small numbers of variables or linear interactions). We aim to extend TE-based effective network inference to *multivariate *techniques, specifically: *capturing *collective interactions where target outcomes are due to multiple source variables (synergies); *eliminating *spurious connections for correlated sources (redundancies); and *avoiding *combinatorial explosions in source groups evaluated. We aim to maximize inference of true interactions while minimizing inference of spurious interactions.

In this manner, we describe a new method [3] which addresses the above requirements in considering multivariate source interactions. For each node in the network, the method identifies the set of source nodes which provide the most statistically significant information regarding its dynamics, and are thus inferred as those source information nodes from which that destination is *computed*. This is done using incrementally conditioned TE, gradually building the set of source nodes for a destination conditioned on the previously identified sources.

**Figure 1:**
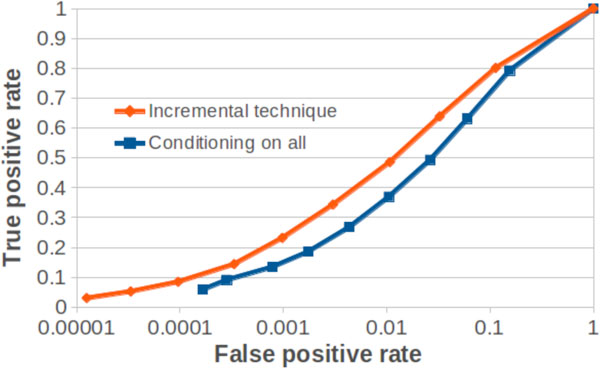
**ROC curve for inference on discrete-time autoregressive time-series for 400 observations (100 nodes, 400 edges, random directed structure, edge weights 0.5, random noise variance 1)**.

For validation purposes, we apply our method to various synthetic models of dynamics on networks (e.g. linearly coupled Gaussian autoregressive time series in Figure 1), and demonstrate the utility of the method in revealing significant proportions of the underlying structural network given only short time-series of the network dynamics. The results show particular utility in comparison to other methods (i.e. Figure 1 shows our incremental method significantly outperforms conditioning on all other variables, even on this data set with linear relationships where the latter is possible). Following validation studies we will apply our technique to neuroimaging data sets.
